# Epidemiology of Coronavirus Disease 2019 (COVID-19) Caused by Severe Acute Respiratory Syndrome Coronavirus 2 (SARS-CoV-2)

**DOI:** 10.1017/dmp.2020.155

**Published:** 2020-05-18

**Authors:** Maoti Wei, Ning Yang, Fenghua Wang, Guoping Zhao, Hongwei Gao, Yuming Li

**Affiliations:** Center of Clinical Epidemiology, TEDA International Cardiovascular Hospital, TianJin, China; Department of Hypertension, TEDA International Cardiovascular Hospital, TianJin, China; Department of Epidemiology, Logistics University of PAP, Tianjin, China; Tianjin Kangting Biotechnology Group Co. Ltd, Tianjin, China and Logistics University of Chinese People’s Armed Police Force (PAP), Tianjin, China; TEDA International Cardiovascular Hospital, TianJin, China

**Keywords:** coronavirus disease 2019 (COVID-19), links, epidemiology characteristics, epidemiology process, epidemic SARS-CoV-2

## Abstract

In December, 2019, an infectious outbreak of unknown cause occurred in Wuhan, which attracted intense attention. Shortly after the virus was identified as severe acute respiratory syndrome coronavirus 2 (SARS-CoV-2), the epidemic of coronavirus disease 2019 (COVID-19) broke out, and an information storm occurred. At that time, 2 important aspects, that is, the stages of spread and the components of the epidemic, were unclear. Answers to the questions (1) what are the sources, (2) how do infections occur, and (3) who will be affected should be clarified as the outbreak continues to evolve. Furthermore, components of the epidemic and the stages of spread should be explored and discussed. Based on information of SARS, Middle East respiratory syndrome (MERS), and COVID-19, the components of the epidemic (the sources, the routes of infection, and the susceptible population) will be discussed, as well as the role of natural and social factors involved. Epidemiologic characteristics of patients will be traced based on current information.

Since the end of 2019, a novel coronavirus, namely severe acute respiratory syndrome coronavirus 2 (SARS-CoV-2), which causes coronavirus disease 2019 (COVID-19), appeared in Wuhan, Hubei Province, China. To date (April 18, 2020), China’s number of officially reported cases include 82,735 confirmed, 48 suspected, 4632 deaths, and 77,062 recovered cases.^1^ The mortality rate caused by COVID-19 in China is 5.60% (4632/82,735; 95% confidence interval [CI]: 5.44–5.76%). At the same time, at least 254,4792 accumulated cases have been reported in nearly all countries and regions outside of China with 175,694 deaths.^2^ To cope with the pandemic, the infection profile caused by the novel coronavirus was summarized from an epidemiological perspective.

## THE EPIDEMIC PROCESS OF INFECTION CAUSED BY SARS-CoV-2

**FIGURE 1:**
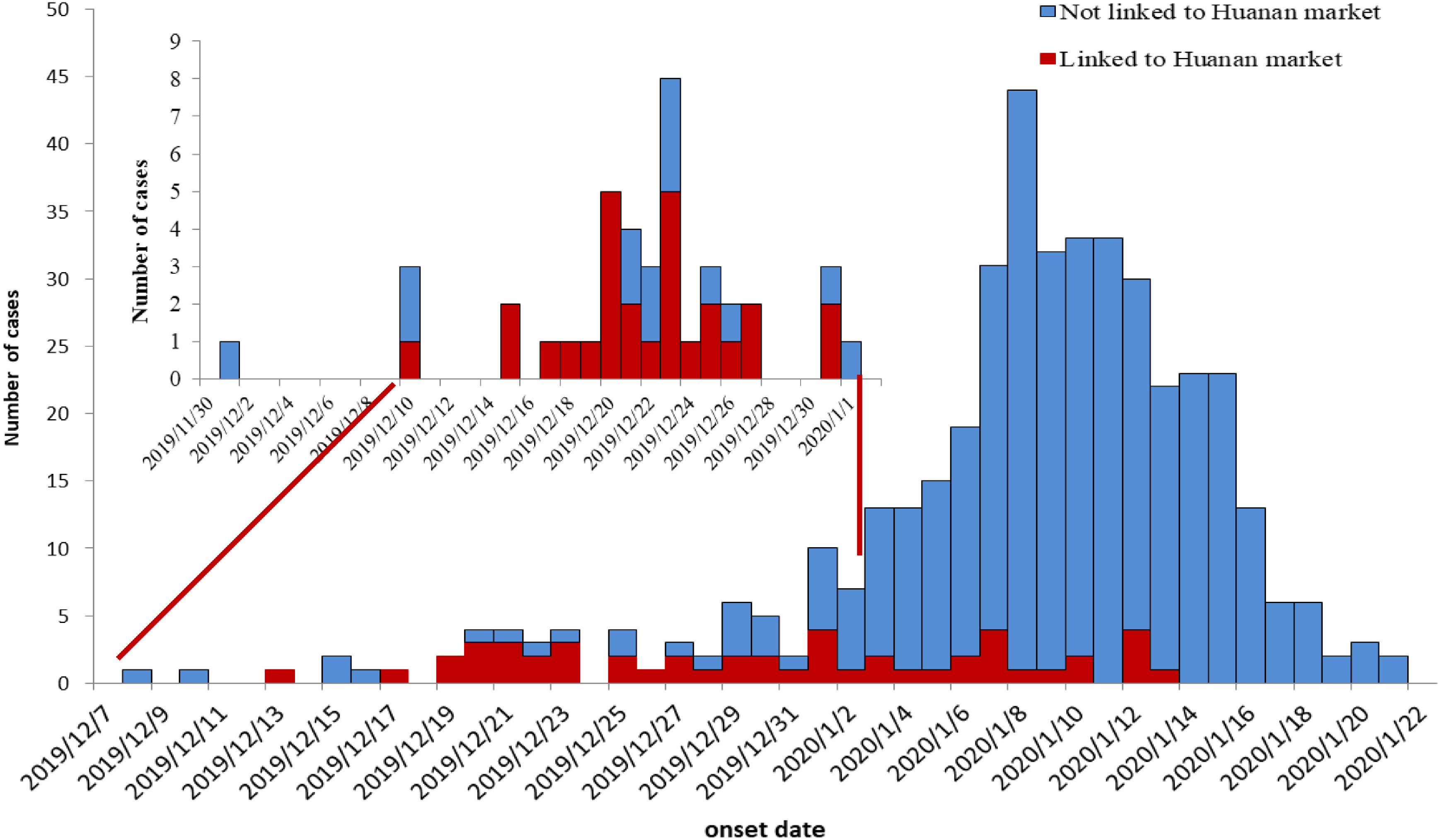
Epidemiological Curve of COVID-19 Cases Reported by Timeline in Published Papers.^3,4^

Note: There is a difference in timing reported by Li et al.^3^ and Huang et al.,^4^ but both studies acknowledged that the epidemic started from mid-December, 2019. The data in the upper litter chart is from Huang et al.,^4^ and the data of the larger chart is from Li et al.^3^

Starting in December 2019, more and more unexplained cases of infection were being found in Wuhan, Hubei Province, China. The initial infection time reported in several studies is different. For example, the study by Li et al. showed that, on December 29, 2019, local hospitals used the term “unexplained pneumonia” for monitoring and found the first reported 4 cases, all of which were related to the South China seafood wholesale market.^3^ The study by Huang et al. reported that 4 patients were found on December 1 and 10, 2019, and 1 patient was related to seafood market exposure.^4^ In all, it was acknowledged that more and more cases were found after mid-December 2019. Published data showed that the progress of the epidemic started from mid-December 2019 with increasing numbers of cases. By December 31, 2019, more than 40 cases (Figure 1, small graph) were reported (Note: there is a slight difference in timing reported by Li et al. and Huang et al., but a similar trend was observed in both studies; that means the epidemic started from mid-December).^3,4^

**FIGURE 2:**
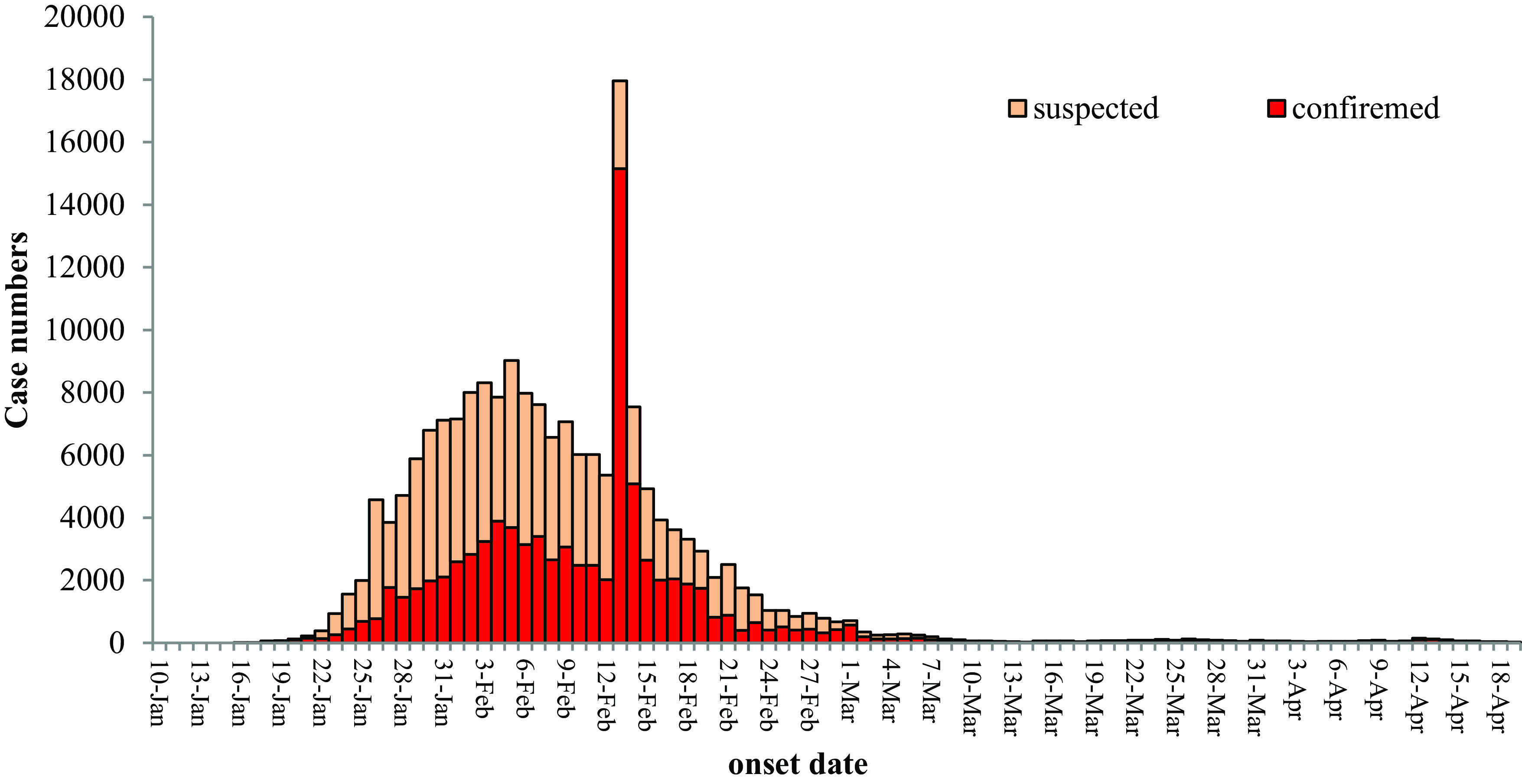
Epidemiological Curve of COVID-19 Cases in China From January 10, 2020, to April 18, 2020, by Daily Reported Data (http://www.nhc.gov.cn/xcs/xxgzbd/gzbd_index.shtml).

Note: From February 5, 2020, the increase trend changed. Shortly after the climax date (February 5, 2020), the extreme spike of cases on February 12, 2020, was explained by the change of diagnosis standard (The Protocols for Diagnosis and Treatment for COVID-19, Fifth Edition, in Chinese), in which clinical cases were included for reporting in Hubei province.

Since the official recognition of human-to-human (person-to-person) transmission, also with the continuous improvement of detection methods and technologies, the number of cases increased rapidly (exponential increase), suggesting that the epidemic is expanding (Figure 2). However, the trend of rising numbers of incident cases changed on February 5, 2020, because strict control measures were enacted and enforced. These data allowed for a profile of the outbreak to emerge. According to daily released data by the National Health Commission (NHC), propagation of COVID-19 could be roughly divided in 4 stages, drawing from the epidemic curve in China.

### Local Outbreak Stage Caused by Seafood Market Exposure

Before the end of December 2019, the epidemic was mainly localized in Wuhan, Hubei Province, China. Most of the cases in this stage were related to exposure to Huanan seafood market. According to a published study, 66% (27/41; 95% CI: 51–80%) of the patients had seafood market exposure.^3^ Although there were many reports of no seafood market exposure, this finding may be explained by limited field epidemiology investigations. Many people may not accurately report exposure history for various reasons.

### Stage of Rapid Expansion of the Epidemic in Wuhan Without Control (or Without Strict Control)

January 1, 2020, to January 23, 2020, was a stage of rapid expansion of the epidemic. During this period, the epidemic spread rapidly from Hubei province to other regions of China. At the same time, the number of cases worldwide was increasing. In this stage, although all levels of the government tried their best to call for disease control in China, disease transmission was inevitable for 3 reasons: first, people were highly mobile due to the coming of the Chinese Lunar New Year celebrations; second, there was a lack of preparedness materials and measures for epidemic prevention at all levels of government; third, information of the epidemic was blurred, and the Wuhan citizens were indifferent to the potential epidemic of infection. For example, a community still held a feast for hundreds of families, even though news of disease transmission was being reported. Those reasons led to the rapid spread of the disease, without control or without strict control.

On January 23, 2020, the National Health Commission (NHC) confirmed 835 cases of infections in 31 provinces (regions, municipalities, including Hong Kong, Macau, and Taiwan). Among them, 177 were in severe status and there were 25 deaths. Thirty-four cases had recovered and were discharged. A total of 1072 suspected cases were reported in 20 provinces (districts and cities). Nine confirmed cases have been reported abroad.^5^

### Rapid Expansion Stage: Out of Control

At noon on January 23, 2020 (Chinese Lunar New Year’s Eve), the prevention and control headquarters of coronavirus infection in Wuhan released announcement No.1, which was an important step to prevent the spread of the epidemic.^6^ The announcement suspended the city’s urban public transportation, subway, ferry, and long-distance passenger transportation, limiting departures from Wuhan, and also temporarily closed the airport and railway station exit channels from the city. Although there were follow-up announcements to limit the spread, with the successful development and distribution of diagnostic reagents in this stage, an exponential growth curve was observed for newly reported cases. At the same time, the World Health Organization (WHO) also declared China as an epidemic area, which has a huge impact on China, and the task of prevention and control became very arduous.

### Lock-Down and Under Control Period

Shortly after the announcement of the lock-down of Wuhan city, the central government initiated several measures for controlling the epidemic in Wuhan as well as in other parts of China (From January 25, 2020, until the time of this writing). Briefly, Wuhan citizens were asked to stay at home, and the government tried its best to offer logistics supplies; medical support was encouraged from other regions to help Wuhan city; infected patients were isolated in Fangcang shelter hospital. As in Wuhan city, other regions of China also carried out strict control measures. The hard work in preventing the epidemic in Wuhan and China paid off, and the incidence cases decreased day by day and a controlled profile was built.

## COMPONENTS OF THE COVID-19 EPIDEMIC

### Source of Infection

#### Wild Animal Source of COVID-19

According to various reports, the initial source of infection is believed to be wild animals, possibly the Chinese bat, *Rhinolophus sinicus.* ^7^ In an interview, academician Zhong Nanshan also mentioned that wild animals, such as bamboo rats and badgers, may also be possible sources. Although there is no correlation between the initial case (December 1, 2019) and the follow-up cases (December 10, 2019), it may be arbitrary to exclude the first patient from the seafood market.^8^ It could take more than 20 days to trace the patient’s contact history in the study; also, the patient’s forgetting or loss of personal contact history is reasonable. Of course, the data also suggest that COVID-19 may have originated far earlier than the time of that patient emerging. The analysis of 27 available genomes of the virus shows that they had a “recent common ancestor” as early as October 1, 2019. It is possible that the first infection case occurred far earlier before December 2019. The virus could have spread among people in Wuhan and other places quietly, and then concentrated and was detected in the seafood market; the virus may have been spreading within the population, and the seafood market may not have been the geographic source.^9,10^

However, the route of transmission to humans at the start of this event remains unclear. Bats are rare in markets in China but are hunted and sold directly to restaurants for food. The current most likely hypothesis is that an intermediary host animal has played an important role in the transmission from animal to human.^8^

#### Patients With Obvious Symptoms as Source of Infection

At present, the existing data and the actual phenomenon of infections showed that COVID-19 patients were the most important source of spread of infection, who could cause human-to-human transmission.^11,12^ When the SARS-CoV-2 infection occurred, it may have been contagious before the appearance of symptoms. However, in SARS-CoV infection, it was reported that the more obvious symptoms were accompanied by a higher viral load, as well as stronger infectivity.^13^ Whether COVID-19 has the same characteristic needs further investigation. Of course, this characteristic also needs close attention in prevention and control. Recent results showed that SARS-CoV-2 persists longer with a higher viral load and peaks later in the respiratory tissue of patients with severe disease; this phenomenon highlights the need for the prevention and control of the epidemic.^14^ Unlike SARS, patients with COVID-19 had the highest viral load near onset of symptom, which could account for the fast-spreading nature of this epidemic.^15^

#### Patients Without Obvious Symptoms or in the Incubation Period

Some experts commented that people with mild or asymptomatic SARS-CoV-2 infection were not identified by epidemic prevention measures, thus accelerating the spread of the disease.^16^ According to published research results, the average interval from onset to the first medical visit was approximately 5–6 days (5.8 days, 95% CI: 4.3–7.5; and 4.6 days, 95% CI: 4.1–5.1), which is similar to the estimated incubation period (the average incubation period of 10 confirmed cases is estimated to be 5.2 days (95% CI: 4.1–7.0).^3^ During that time, although there were mild or no symptoms, the disease could be contagious. This finding highlights the importance of contact history tracing and maintained isolation of patients, suspected patients, and those with whom they have come in contact. This feature of COVID-19 is significantly different from that of the SARS-CoV infection outbreak in 2003. It is reported that SARS had no evidence of an incubation period and no shedding of virus.^13^

Asymptomatic patients were reported often, and this meant that there may be so-called “recessive infection cases” or asymptomatic infections.^17,18^ Most of the so-called “recessive infection cases” or asymptomatic infected people would eventually have symptoms and become ill.

#### Super Spreader

To date, no super spreader of the SARS-CoV-2 infection has been observed, but experts still warned people to pay attention to the possibility of super transmission events. Clustering cases were reported, but in limited scale.^11,12^ These studies reported that a super spreader at a group gathering could cause 5–6 new cases of infection, with a maximum of 7 people newly infected being reported. However, compared with SARS-CoV infection, there were fewer clustered cases of SARS-CoV-2 infection and fewer cases compared with Middle East respiratory syndrome coronavirus (MERS-CoV) infection.^19^ However, other data showed that the transmission ability of COVID-19, with an R_0_ = 2.2 to 2.68, indicates that there may be super spreaders of the virus.^3,20^ For effective prevention of mass transmission, more must be learned about super spreaders of COVID-19.

### Transmission Routes

#### Nearby Droplet Propagation

Inhalation of droplets containing virus particles exhaled by infected persons is the main mode of transmission of COVID-19, and the most common route of infection. This mode of transmission is very similar to that of the SARS-CoV infection. This transmission is easy to achieve during daily contacts, such as close family contact, by public transportation, in medical institutions, and so on. The reported COVID-19 cluster events may be explained by close family contact.^3,11,12^ It is generally believed that large size droplets play important roles in SARS spread, especially droplets with sizes of 5 μm-10 μm; so how about COVID-19? Because the symptoms of COVID-19 are very similar to those of influenza or the common cold, they are easily misdiagnosed in the early stages of infection by clinicians, other medical staff, or rescue personnel. In the early stages of infection, cutting off the spread of droplets may play an important role in the control of COVID-19 spread.

During the epidemic of SARS, aerosol transmission was 1 way of transmission, which was highly suspected to be 1 of the important transmission routes of outbreaks in hospitals and communities in severely affected epidemic areas. The epidemiological point here is that susceptible persons can be infected by inhalation of aerosols containing SARS-CoV without coming into contact with SARS-infected patients. Although the data of SARS cases were analyzed by epidemiological models, the results showed that the spread of SARS infection was not supported by aerosols, but by droplets. Officers of WHO believed that the possibility of SARS transmission through aerosols is very low. However, the propagation speed of COVID-19 is so fast, whether it is related to aerosol propagation needs further confirmation. However, the respiratory transmission will be greatly reduced by various protective measures, such as wearing face masks and isolation clothing.

#### Close Contact Transmission

Through direct or indirect contact with the patient’s secretions or body fluids, excreta, and other contaminated items, or working for, living with, treating, or visiting the patient, etc., pathogens may invade the body through the mouth, nose, and eye mucosa, to achieve transmission. During the epidemic period of SARS, close contact is another important way of transmission. Whether COVID-19 also depends on this mode of transmission needs further confirmation. However, current data have confirmed that close contact during living together, working, or diagnosis and treatment can result in transmission.^5,12,17^

#### Other Transmission Routes

During the course of the SARS epidemic, the possibility of transmission by means of the digestive tract cannot be ruled out because of lack of direct evidence. However, a study reported that there were digestive tract symptoms after COVID-19; whether the transmission was achieved through the digestive tract was unknown and needs further epidemiological study.^21^ To date, it is confirmed that SARS-CoV-2 viral RNA can be detected in feces after recovery, which is very similar to the SARS-CoV infection.^22^ It is unknown whether the virus is infectious at that time. However, enhanced control measures of the digestive tract products may reduce the risk of its spread during the epidemic.

To date, there is no evidence of transmission by blood, sexual contact, vertically from mother to fetus, or other routes. There is no evidence that flies, mosquitoes, cockroaches, and other arthropods can transmit COVID-19.

### Susceptible Populations

People are generally susceptible to infection by SARS-CoV, but the rate of infection in children is low, and the reason is unclear. Some experts concluded that children are not easily infected. However, epidemiological data show that children are susceptible to COVID-19, and the low infection rate of children may be related to their having less exposure.^3^ The elderly and those with underlying diseases are seriously affected after infection. Preliminary data showed that most patients who die are the elderly with underlying diseases.

Those persons in close contact with SARS patients during the symptomatic period are at high-risk for SARS-CoV infection. However, persons coming in contact with an asymptomatic COVID-19 patient may also be at high-risk of SARS-CoV-2 infection. Persons caring for and visiting patients (medical staff, patients’ family members and friends) were likely to have close and prolonged contact times. If sufficient protective measures were not taken, these persons are at higher risk of acquiring COVID-19.

It is confirmed that humoral immunity can be induced after SARS-CoV infection. The serum anti SARS-CoV immunoglobulin (Ig) G stays strongly positive at 6 months after the onset of the disease.^13^ For COVID-19, whether the humoral immunity could have protective effects needs to be studied further. In the acute period of SARS-CoV-2 infection, the level of some cytokines increased: interleukin (IL) -1B, IL-1RA, IL-7, IL-8, IL-9, IL-10, basic fibroblast growth factor (FGF), granulocyte colony-stimulating factor (GCSF), granulocyte-macrophage colony-stimulating factor (GMCSF), interferon-gamma (IFN-γ), induced protein-10 (IP-10), monocyte chemoattractant protein-1 (MCP1), macrophage inflammatory protein-1A (MIP1A), MIP1B, platelet-derived growth factor (PDGF), tumor necrosis factor-alpha (TNF-α), and vascular endothelial growth factor (VEGF). However, there was no significant change in other cytokines, such as IL-5, IL-12p70, IL-15, eotaxin, and RANTES.^3^ Certainly, these altered cytokines may be related to the development of this disease. A recently published study showed that the elevated IL-6 concentration could be associated with poor prognosis.^15^ Different from SARS-CoV infection, COVID-19 can cause severe cellular immune dysfunction.

### Natural and Social Factors on the Epidemic of COVID-19 Infection

#### Natural Factors

According to data of respiratory diseases, poor ventilation, environmental conditions, and mass gathering indoors will favor the transmission of infectious diseases. Some person-to-person transmissions may be explained by closed environments.^11,12^ As is well known, the SARS outbreak ceased with the coming of the summer, and it seems there is a direct relationship between the seasonal factors and the spread of this coronaviruses infection among human beings. Therefore, further investigated is needed as to whether the epidemic of COVID-19 relates to meteorological conditions, seasonality, geographical conditions, and ecological environment. The pandemic of COVID-19 occurred in the northern hemisphere and the southern hemisphere, and this fact means that infections of SARS-CoV-2 may not be influenced by temperature.

#### Social Factors

High population density, high mobility, poor health conditions, and poor health habits are important aspects prompting the spread of infectious diseases. Large cities with concentrated populations and convenient transportation are prone to outbreaks and the prevalence of respiratory infectious diseases. Ineffective prevention and control measures and improper personal hygiene habits and protective measures of medical staff may cause the occurrence of nosocomial infections. The rapid spread of SARS is closely related to mass migrations using modern transportation. The prevalence of COVID-19 increased rapidly in a short period, also demonstrating that current transportation modes are crucial for the spread of this disease. Therefore, it is very important to cut off all responsible transmission routes (through various means) once the transmission routes of the disease are well defined.

According to established prevention and control of infectious disease strategies, areas may be declared epidemic areas after an outbreak of an infectious disease, and corresponding control measures enacted. After the outbreak of COVID-19, Wuhan city has been closed (locked down) since January 23, 2020, and the control measures have been in effect from February 5, 2020. Also, according to a study, “close the city” has reduced the incidence of infection and death by nearly 70%.^23^ In addition, the state vigorously publicized the epidemic control measures, which also played an important role in the control of COVID-19 in China.

With the ending of the Spring Festival holiday, people began to return home to their cities (city-returning). There were 4 relatively large zones where people were returning to, namely Beijing, Tianjin, and Hebei; the Yangtze River Delta; the Pearl River Delta; and Chengdu and surrounding areas. It was previously predicted that, with the large number of people moving back to specific areas, the number of new infections would also increase. Therefore, the epidemic could worsen because of the breakdown of the prevention and control measures. Fortunately, at the time of this writing, the epidemic of the COVID-19 is under control in these 4 regions, as well as in China.

## EPIDEMIC OF COVID-19

### Epidemic in China

On April 18, 2020, the National Health Commission (NHC) had received 82,735 total confirmed cases reported by 31 provinces (autonomous regions, municipalities directly under the central government) and Xinjiang production and Construction Corps, including 1041 confirmed (85 in serious condition), 4632 deaths, 77,062 discharged cases, and 48 suspected cases.^1^

**FIGURE 3:**
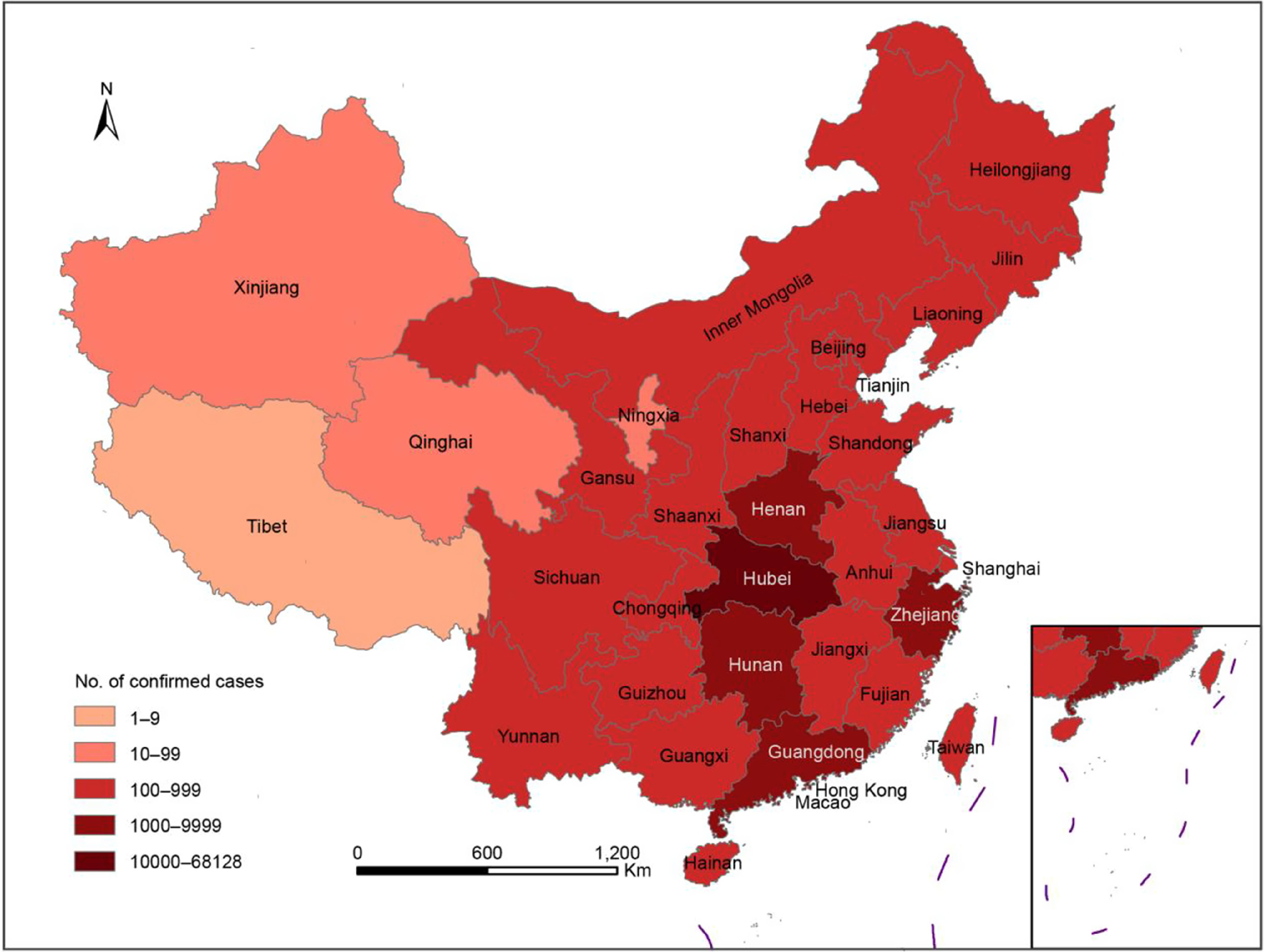
Regions With Total Reported Confirmed Cases of COVID-19, China, Until 18 April 2020 (http://www.nhc.gov.cn/xcs/xxgzbd/gzbd_index.shtml).

The hottest epidemic area in mainland China was Wuhan city, Hubei Province, followed by Guangdong Province, Henan Province, Zhejiang Province, Hunan Province, and so on. On the whole, infections were more likely in areas surrounding Hubei province (Figure 3).

### Temporal Distribution

#### The Epidemic of COVID-19 Outside China in the Early Period

From the first case in Wuhan in November 2019 to the first case of COVID-19 in Thailand on January 8, 2020 (the first case outside China), COVID-19 had successively appeared in other countries. With the spread of the epidemic, it was listed as a public health emergency of international concern (PHEIC) by WHO on January 30, 2020.

**FIGURE 4:**
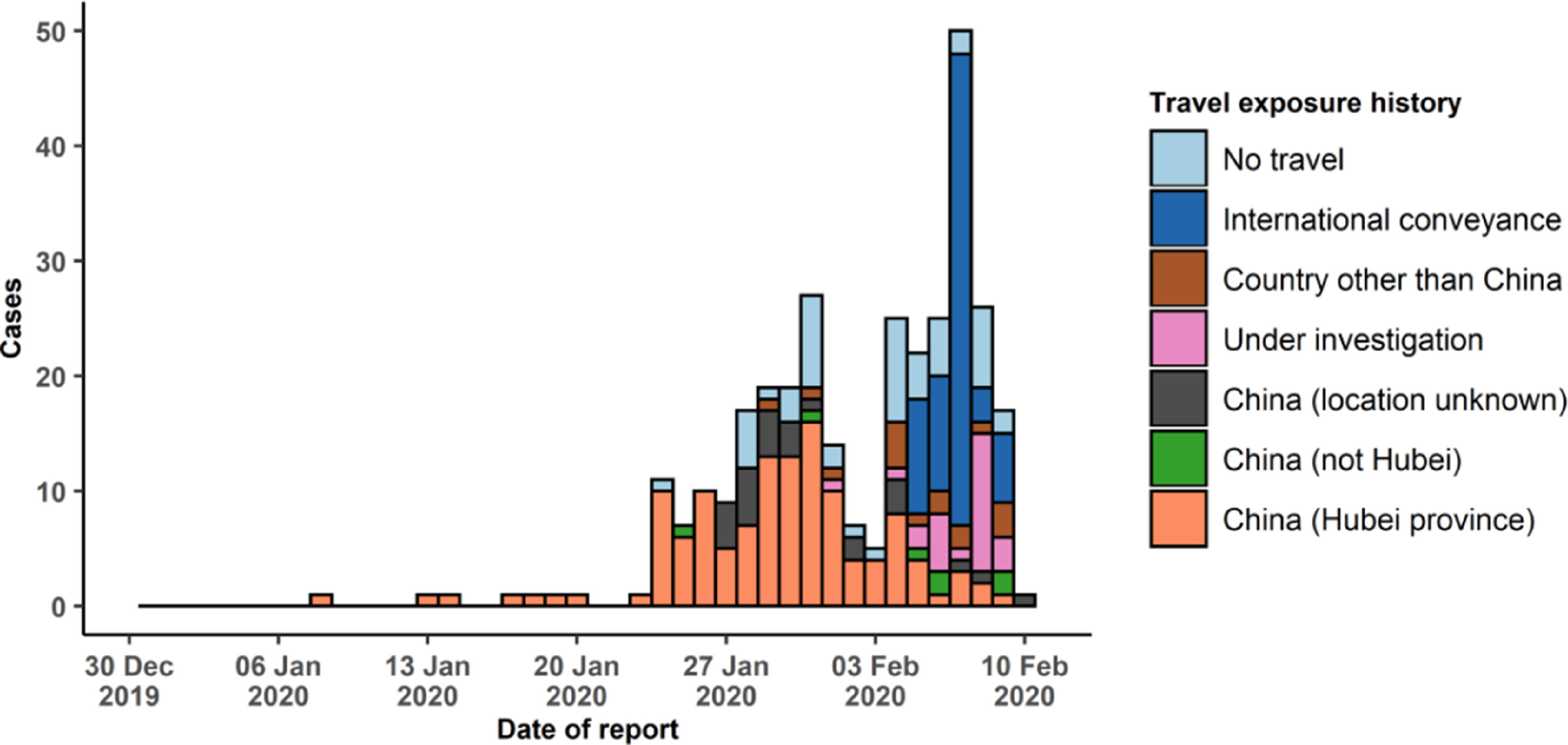
Epidemic Curve of COVID-19 Cases (*n* = 319) Identified Outside of China, by Date of Reporting and Travel History, February 10, 2020.^23^

The epidemic curve of COVID-19 worldwide showed that early cases occurred sporadically, but the number of cases increased significantly after February 10, 2020 (Figure 4). The mode of infection increased gradually from the visit to Wuhan to the contact with the residence. As shown in the data, most of the cases were initially exposed to Hubei (or Wuhan), or were related to taking transportation or going to Hubei (or Wuhan).^24^ However, some cases in the later period were related to contact with patients.^11^ At the time of this writing, the pandemic is roaring all around the world.

#### The Epidemic of COVID-19 in China

Official reports showed a rapid increase in cases of COVID-19 was observed in the epidemic curve. With the coming of the city-returning peak (people returning to their home and work city after the Spring Festival holiday), it was warned that more cases would be reported in other places of China. An epidemic curve is presented (Figure 2), in which, an increase trend in the early course and a down trend in the later period. Although some studies thought that numbers of cases were seriously underestimated, fortunately the down trend has maintained, and this means the epidemic is under control in China.^20^

### Epidemiological Characteristics of COVID-19

#### Occupation, Age, and Gender

During the early period of the epidemic, limited data were obtained for describing the epidemiological characteristics of COVID-19. However, according to 2 published studies, the source of infection was located to the Huanan seafood market. For this reason, the personnel related to the seafood market could be roughly divided into 3 categories: the first category was the dealers and the managers of the seafood market (who had close contact with the seafood market); the second category was the buyers at the seafood market (who had contact history with the seafood market); the third category was the secondary cases of the infections in the seafood market (who had no contact history with the seafood market).^3,4,25^

**FIGURE 5:**
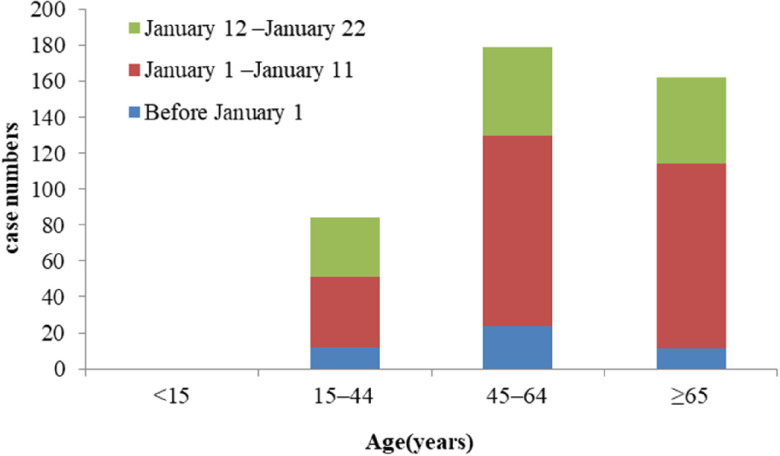
Age Distribution of 425 patients With COVID-19 (Revised From Data of Li et al.^3^).

Among the 425 patients reported by Li et al., the first time period was before January 1 (the date of closing the Huanan seafood wholesale market). The average age of 47 patients was 56 years (26–82 years), and 77% of them were under 64 years old.^3^ According to the common assumption, the first group (the dealers or the managers of the seafood market) should account for a large part of the affected population in this period, and there were no children under the age of 15 at this stage. In the period after the closure of the seafood market, ie, the second and third stages in the study by Li et al., 3 groups of people (close contact, contact, and no contact with the seafood market) may exist. At this time, the average age of patients was 60 years (15–89 years old) (Figure 5). However, SARS-CoV-2, as a new virus, the people with the highest risk were those people with low resistance, such as the elderly, especially the elderly with underlying diseases, where infections may be more severe. That means the weak more easily contracted infection, with more serious outcomes. This phenomenon has been observed in other binfluenza virus infections, such as the H7N9 virus, which affected the elderly at a higher significantly rate than other common pathogens.^26^

Therefore, it could be preliminarily considered that the main personnel before closure of the market were the dealers or the managers of the seafood market, the purchasers at the seafood market, and the secondary cases of the infected persons in the seafood market. After the closure of the market, onsets of the early stage of infections and secondary transmissions were more likely to affect the elderly with underlying diseases. Therefore, in the follow-up prevention and control of the epidemic, more attention should be paid to the elderly, especially the elderly with underlying diseases; high death rates reported verified this hypothesis now.

Data showed that the proportion of men in the first period was higher (66%); in the second period, the proportion of men decreased (59%); and in the third period, the proportion of men was almost the same as women (48%). This phenomenon confirms the stronger relationship with age; in other words, there was no significant gender difference in COVID-19. This point was confirmed by WHO published data with 716,570 cases reported on April 18, 2020.^2^

Regarding occupational characteristics, the proportion of medical staff in the cases changed gradually in the 3 periods. It is suggested that, in the process of fighting against the epidemic, the medical staff had a great risk of infection, so it is necessary to ensure the medical equipment of the front-line medical staff. Basic preventive measures, such as wearing masks, gloves, washing hands frequently, and wearing isolation clothes, can stop the spread of the disease. With the spread of the pandemic of COVID-19 out of China, more and more medical staffs were infected, especially for the countries not well prepared. Therefore, it is urgent to strengthen preparedness for medical staff by ensuring adequate amounts of proper equipment for every country.

### The Effect of Different Comorbidities on the Prognosis of COVID-19

During the epidemic of SARS, data showed that suffering from underlying diseases can affect the prognosis of SARS patients. The mortality rate of SARS in China is 6.57%, and the proportion of the elderly is relatively large (44% of were over 60 years old). With increased age, the fatality rate also increased. The patients with other diseases, such as hypertension, diabetes, heart disease, emphysema, and tumor, had the highest fatality rates.

On February 2, 2020, the number of deaths due to COVID-19 reported by the state was 304, with a mortality rate of 2.11% (304/14380), which was lower than that of SARS-CoV and MERS-CoV infection. According to the current limited data (17 case fatalities), the average age of the patients was 73 ± 12 years (48–89 years), and most of them had underlying diseases.^27^ Although the data were not very comprehensive, it was speculated that the prognosis characteristics may be similar to that of SARS-CoV infection.

In conclusion, in view of the infection caused by the COVID-19, this study used the research literature, data, and daily releases to summarize the epidemic process and the transmission stages of COVID-19. The infection source, transmission routes, susceptible populations, and natural and social factors affecting the epidemic of COVID-19 were summarized. The epidemiological characteristics of COVID-19 were analyzed and described. Through this study, we hope to preliminarily clarify the epidemiology of infections caused by the new coronavirus and provide guidance for prevention and control.
